# Retinal astrocytic hamartoma complicated by branch retinal vein occlusion in a patient with tuberous sclerosis complex

**DOI:** 10.1016/j.ajoc.2023.101920

**Published:** 2023-08-24

**Authors:** Pedram Afshar, Haniyeh Zeidabadinejad, Fariba Ghassemi, Hamid Riazi-Esfahani, Elias Khalili Pour

**Affiliations:** Retina Ward, Farabi Eye Hospital, Tehran University of Medical Sciences, Tehran, Iran

**Keywords:** Retinal astrocytic hamartoma, Branch retinal vein occlusion, Tuberous sclerosis complex

## Abstract

**Purpose:**

To report a case with branch retinal vein occlusion secondary to a retinal astrocytic hamartoma in a patient with tuberous sclerosis complex.

**Observations:**

A fourteen-year-old boy, a known case of tuberous sclerosis complex, with multiple bilateral retinal astrocytic hamartomas was followed by 6 months intervals. In his last follow-up, 6 months after initial presentation, the patient developed angiographic signs of branch retinal vein occlusion (BRVO) in the superotemporal arcade of the right eye distal to one of the retinal astrocytic hamartomas. He underwent targeted retinal laser photocoagulation. No secondary complication related to BRVO was observed during the next six-month follow-up.

**Conclusion:**

And Importance: Although the co-occurrence of branch retinal vein occlusion and astrocytic hamartoma may represent an incidental finding, awareness of BRVO as a possible complication associated with retinal astrocytic hamartoma helps timely diagnosis and prompt treatment of this complication, improving the visual prognosis of these patients.

## 1 Introduction

The ocular manifestations of Tuberous Sclerosis Complex (TSC), an autosomal dominant multisystem disorder, include retinal astrocytic hamartomas, achromatic retinal patches, and, less frequently, iris and ciliary body pigment epithelium hamartomas.^1^, ^2^, ^3^ Retinal astrocytic hamartomas have been reported in one-third to one-half of TSC patients.^4^ They are considered relatively quiescent lesions which rise from retinal glial cells with little potential for progression. However, in rare situations, they may reveal aggressive behaviors including rapid growth.^5^, ^6^, ^7^ Numerous ocular complications of retinal astrocytic hamartomas have been documented, including vitreous hemorrhage, and retinal vascular abnormalities such as neovascularization, telangiectasia, exudation, and vitreous seeding.^8^, ^9^, ^10^ The objective of this case was to document an infrequent occurrence of branch retinal vein occlusion (BRVO) that coincided with retinal astrocytic hamartoma in a patient diagnosed with TSC.

## 2 Case report

**Fig. 1:**
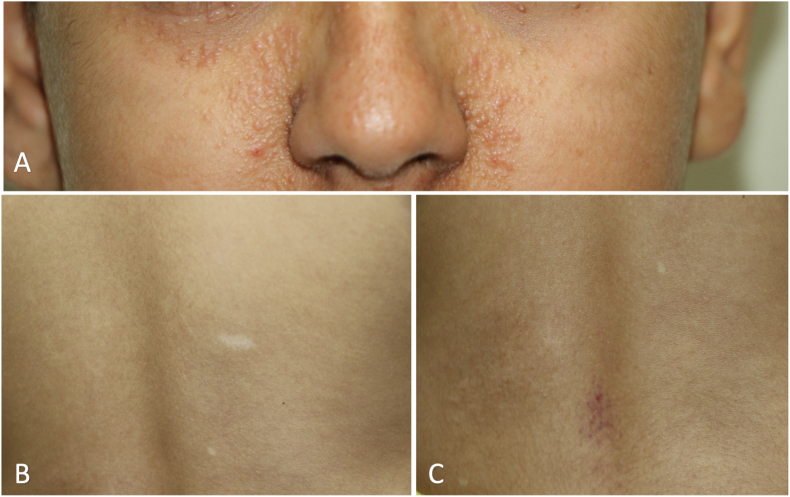
(A) facial angiofibromas, formerly called as adenoma sebaceum (B) ash-leaf sign, hypopigmented macule and patch and (C) shagreen patch, flesh-colored leathery fibromatous skin infiltration on lumbosacral area.

A fourteen-year-old boy (known case of tuberous sclerosis complex) was referred to our ophthalmology department for an ophthalmic examination. He was unable to communicate verbally due to his intellectual disability. Skin examination revealed facial angiofibroma, ash-leaf lesions on the trunk, and shagreen patches (Fig. 1A–C). He was unable to cooperate for visual acuity. His refraction was “Plano-1.25 × 180” in his right eye and “Plano-0.50 × 180” in his left eye. He had normal intraocular pressure and unremarkable anterior segment slit-lamp examination in both eyes.

**Fig. 2:**
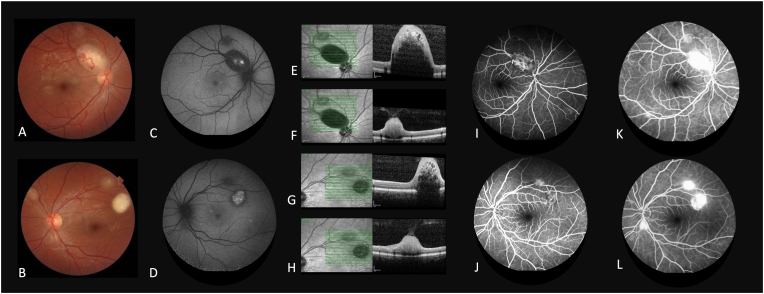
Baseline fundus photography of the patient revealed multiple bilateral lesions compatible astrocytic hamartomas (A, B). In the right eye, a large elevated peripapillary lesion along with the superior vascular arcade with 2-disc diameter size and internal calcified areas was seen. Also, a flat-type tumor superior to the vascular arcade sizing about two third of the disc diameter was found in the right eye (A). In the left eye, three lesions were visible (B). On fundus autofluorescence (FAF) imaging of the right and left eye (C, D), the large superotemporal astrocytic lesion of the right eye displayed hyperautofluorescent core with surrounding hypoautofluorescent area (C). On spectral-domain optical coherence tomography (SD-OCT) and corresponding Infrared reflectance images of the astrocytic hamartomas (E–H), hyporeflective areas surrounded by hyperreflective areas with variable amounts of posterior shadowing and abnormal vitreoretinal interfaces, consistent with retinal astrocytic hamartomas was visible. Intra-retinal cystic spaces which gave them a moth-eaten appearance were visible in the superficial portions of hamartomas with calcific components (E and G). Early-phase fluorescein angiography (I and J) revealed mild hypofluorescent regions caused by tumor blockage. During the late venous phase, leakage from astrocytic lesions caused hyperfluorescence over the tumors (K and L).

Posterior segment examination revealed multiple bilateral creamy-white appearance lesions which were compatible with astrocytic hamartomas (Fig. 2A and B). In the right eye, a large elevated peripapillary lesion with a 2-disc diameter size was seen with internal calcified areas, along with the superior vascular arcade. Also, a flat-type lesion superior to the vascular arcade sizing about two third of the disc diameter was found in the right eye. In the left eye, three lesions were visible. An elevated calcified lesion measuring about 1.5-disc diameters in the superior macular area. A flat lesion measuring two third disc diameter was seen along with the superior vascular arcade. A third flat lesion one disc diameter in size was observed superonasal to the optic disc.

On fundus autofluorescence (FAF) (Spectralis HRA + OCT®, Heidelberg Engineering Inc., Heidelberg, Germany) imaging of the right eye, the large superotemporal astrocytic lesion displayed a hyperautofluorescent core with surrounding hypoautofluorescent area. The superior lesion to the above-mentioned astrocytic hamartoma showed uniform hypoautofluorescence. In the left eye, FAF revealed a large astrocytic lesion superotemporal to the fovea with central hyperautofluorescence, and a hypoautofluorescence rim, along with a smaller hypoautofluorescent lesion next to the larger lesion (Fig. 2C and D).

Spectral-domain optical coherence tomography (SD-OCT) (Spectralis HRA + OCT®, Heidelberg Engineering Inc., Heidelberg, Germany) through the lesions was performed. All tumors were confined to the inner retinal layers and typically appeared as dome-shaped retinal thickening with disruption of the normal layers. Compatible with retinal astrocytic hamartomas, these lesions featured hyporeflective centers surrounded by hyperreflective periphery, varying degrees of posterior shadowing, and abnormal vitreoretinal interfaces.^1^ Intra-retinal cystic or cavital spaces which gave them a moth-eaten appearance were visible in the superficial portions of hamartomas with calcific components. No macular edema was seen bilaterally (Fig. 2E–H).

Early-phase fluorescein angiography (FA) (Spectralis HRA + OCT®, Heidelberg Engineering Inc., Heidelberg, Germany) revealed mild hypofluorescent regions caused by tumor blockage. During the late venous phase, leakage from astrocytic lesions caused hyperfluorescence over the tumors (Fig. 2I–L).

**Fig. 3:**
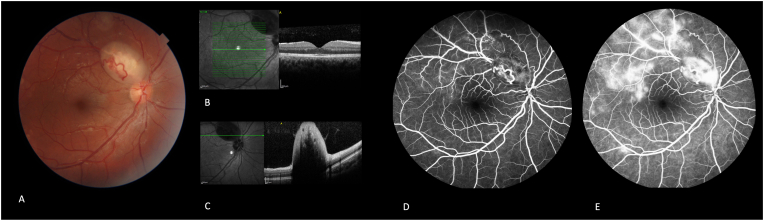
Fundus examination was conducted six months after initial presentation, which revealed a slight venous engorgement located distal to the superotemporal astrocytic hamartoma in the right eye. (A). The SD-OCT of the right eye through the fovea revealed no evidence of macular edema (B) and OCT through the superotemporal astrocytic lesion shows hyporeflective centers surrounded by hyperreflective periphery, varying degrees of posterior shadowing, and abnormal vitreoretinal interfaces (C) and Early phase fluorescein angiography of the right eye revealed hyperfluorescent areas along the superior retinal vein distal to the tumor (D), which in the late phase had increasing perivascular hyperfluorescence due to leakage along the branches of the superior vascular arcade (E). This was consistent with branch retinal vein occlusion along with the superior vascular arcade.

Follow-up visits every six months were planned. Fundus examination was conducted six months after initial presentation, which revealed a slight venous engorgement located distal to the superotemporal astrocytic hamartoma in the right eye (Fig. 3-A). The SD-OCT and FA of the right eye revealed no evidence of macular edema or nonperfusion areas (Fig. 3B and C).

FA of the right eye in the early phase revealed hyperfluorescent areas along the superior retinal vein distal to the tumor, which had increasing hyperfluorescence due to leakage in the late phases (Fig. 3D and E). This was consistent with BRVO along with the superior vascular arcade.

**Fig. 4:**
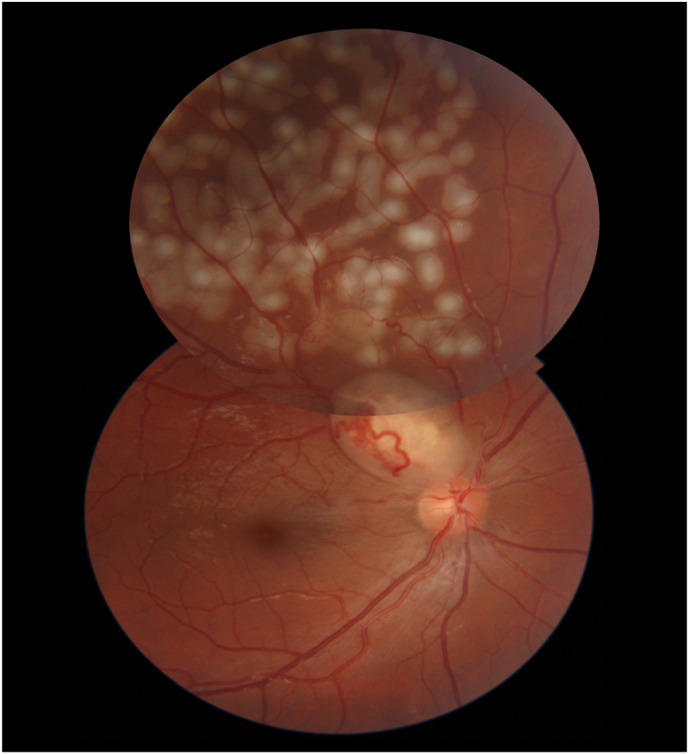
Fundus photography of the patient after targeted retinal laser photocoagulation.

In the left eye's FA, no new vascular abnormality was observed. The patient had targeted laser photocoagulation of the retina in regions of leakage (Fig. 4). At the six-month follow-up, he was clear of complications associated with BRVO; nevertheless, he is being monitored for adverse events such as retinal or iris neovascularization.

## 3 Discussion

This case report presents a patient with TSC who presented with an extremely unusual ocular manifestation, an astrocytic hamartoma accompanied by BRVO. After conducting a literature review in April 2023 utilizing PubMed, Google Scholar, and Scopus (using the key words: “tuberous sclerosis complex”, “tuberous sclerosis” “astrocytic hamartoma” “astrocytoma” and “Retinal Vein Occlusion”), we did not find any similar report.

Bourneville initially characterized TSC in the 1880s. It is a multisystem, autosomal dominant condition caused by mutations in either TSC1 (encoding hamartin) or TSC2 (encoding tuberin).^2^ TSC2 mutations (75–80%) are more prevalent than TSC1 mutations (10–30%) and are associated with a greater incidence of retinal abnormalities.^11^^,^^12^ TSC frequently causes a wide variety of neurologic disorders, such as intellectual disability, epilepsy, and autism, in addition to cutaneous manifestations such as facial angiofibroma, formerly known as adenoma sebaceum, shagreen patches, hypomelanotic macules (ash-leaf lesions), and ungual fibromas. Brain subependymal giant-cell tumors, kidney cysts or angiomyolipoma, pulmonary lymphangiomatosis, and cardiac rhabdomyomas are among the most common manifestations of this condition.^2^^,^^13^^,^^14^

In patients with TSC, many ocular manifestations have been recorded, including retinal astrocytic hamartomas, achromatic retinal patches, and, less frequently, iris and ciliary body pigment epithelium hamartomas.^3^ Retinal abnormalities are related with epilepsy, cognitive impairment, renal angiomyolipoma, and subependymal giant cell astrocytoma with greater probability.^12^ Approximately one-third to one-half of TSC patients have benign retinal astrocytic hamartomas, according to prior studies.^4^ However, incidence as high as 87% have been reported.^15^ According to the study by Aronow et al. retinal astrocytic hamartomas were bilateral in 43.3% of patients and multiple in 40% of patients when they were present. The majority of these lesions are found in the posterior pole, along the vascular arcades, and close to the optic nerve.^12^ Retinal astrocytic hamartomas are morphologically classified as a flat, translucent, non-calcified lesion (type 1), a whitish nodular mulberry-like calcified lesion (type 2), and an intermediate transitional lesion that is calcified in the central portion and semi-translucent at the periphery (type 3).^4^^,^^16^ They are the best-known ocular manifestation of TSC but can be idiopathic or associated with neurofibromatosis.^17^ According to the morphological classification of astrocytic hamartomas and the concomitant BRVO in the present case, type 2 lesions appear to be more accountable for this complication.

OCT of astrocytic hamartomas typically reveals a dome-shaped hyperreflective mass arising from the retinal nerve fiber layer in conjunction with variable retinal disorganization, posterior shadowing, and intralesional optically empty spaces (OES), appearing as so-called moth-eaten spaces, representing intralesional calcification or cavitation.^18^^,^^19^ Based on spectral domain optical coherence tomography (SD-OCT), Pichi et al. divided retinal astrocytic hamartomas into four categories and revealed systemic TSC correlations with each class.^20^ Type I exhibited SD-OCT findings of a flat lesion and clinical findings of no retinal traction. Type II demonstrated SD-OCT evidence of a slightly raised (height <500 μm) lesion as well as clinical indications of retinal traction. SD-OCT revealed an increased retinal mass (height >500 μm) with inner retinal calcification and clinical indications of mulberry-like calcification in Type III. Type IV exhibited SD-OCT evidence of a raised retinal mass (height >500 μm) and an optically empty hollow, as well as clinical indications of a smooth, noncalcified inner retinal mass. According to the OCT-based classification of astrocytic hamartomas and the concomitant BRVO in the present case, type III lesions appear to be more accountable for this complication.

Depending on their calcium content, astrocytic hamartomas exhibit hyperautofluorescence on FAF imaging.^21^

On FA, early blockage owing to the tumor mass is seen during the choroidal phase, when the intrinsic tumor vasculature begins to fill, and hyperfluorescence gradually rises throughout the arterial phase. A possible leakage may occur in the late venous phase.^22^

Multiple ocular consequences of astrocytic hamartomas of the retina have been observed. These conditions include vitreous hemorrhage, retinal vascular anomalies including neovascularization, telangiectasia, and exudation, and vitreous seeding.^8^, ^9^, ^10^ In the current case report, we described an extremely uncommon complication of retinal astrocytic hamartoma. A BRVO occurred in the region of a retinal astrocytic hamartoma in our patient. An eye with an epipapillary astrocytic hamartoma was reported to have a branch retinal artery obstruction (BRAO).^23^

The mechanism of vascular occlusion in astrocytic hamartoma-affected eyes remains unknown. As Vedantham et al. also mentioned in their BRAO study, the pathophysiology of venous blockage in our case may be owing to increased resistance and therefore delayed circulation in draining veins, which increases the likelihood of vascular obstruction.^23^ Endothelial damage of tumor vasculature may result in thrombosis, and vascular blockage may ensue due to increased hydrostatic pressure in the retinal veins in the astrocytic hamartoma region, according to a second proposed explanation.^24^

In this case, we preferred to do targeted laser photocoagulation of the retina in regions of leakage to reduce the amount of vitreous vascular endothelial growth factor (VEGF). Previous studies have shown that astrocytic hamartomas, especially in patients with TSC, may be intrinsically associated with VEGF signaling for their structure and exudative properties independent of their ability to promote neovascularization.^8^^,^^25^^,^^26^ Some case reports exhibit clinical improvement in response to *anti*-VEGF therapy without requiring any evidence of pre-retinal or choroidal neovascularization.^26^, ^27^, ^28^ As vein occlusions can cause VEGF overproduction, avoiding to do proper management may cause tumor growth and secondary mentioned complications.^29^

In light of the patient's cognitive impairment and geographical distance from the retina clinic, targeted retinal photocoagulation was deemed the preferred treatment option. It is not possible to assert that targeted laser photocoagulation represents the optimal treatment modality for this particular patient. Rather, the selection of treatment options should be tailored to the individual patient, taking into account factors such as the risk of loss to follow-up and the stratification of treatment options, such as the potential risks associated with intravitreal injection in a patient with mental retardation.

The approach that was employed in the present case has specific limitations. The presence of localized vasculitis-like appearance in the superior arcade of the right eye warrants consideration of inflammatory processes that may be secondary to conditions such as sarcoidosis or tuberculosis. In cases where there is concomitant astrocytic hamartoma and vasculitis-like appearance, it is advisable to conduct inflammatory workups in this group of patients. Alternatively, employing a multimodal imaging strategy, such as optical coherence tomography angiography (OCTA), may aid in the identification of non-perfused regions in the current patient, thereby necessitating additional assessments and interventions. And finally, the patient was identified as a known case of tuberous sclerosis complex; however, due to the absence of genetic testing, the pathogenic variant of the patient could not be determined. As a result, it is unclear whether the patient belongs to the TSC1 or TSC2 variants of tuberous sclerosis complex.

## 4 Conclusion

Although the co-occurrence of BRVO and astrocytic hamartoma may represent an incidental finding, awareness of BRVO as a possible complication associated with retinal astrocytic hamartoma helps timely diagnosis and prompt treatment of this complication, improving the visual prognosis of these patients.

## Patient consent

Written informed consent was obtained from the patient's family for publication of this case report and the accompanying images.

## Funding

No funding or grant support.

## Authorship

All authors attest that they meet the current ICMJE criteria for authorship.

## Declaration of competing interest

The authors declare that they have no known competing financial interests or personal relationships that could have appeared to influence the work reported in this paper.
